# Use of Professional Interpreters for Patients With Limited English Proficiency Undergoing Surgery

**DOI:** 10.1001/jamanetworkopen.2023.55014

**Published:** 2024-02-06

**Authors:** Jenny Cevallos, Carmen Lee, Tasce Bongiovanni

**Affiliations:** 1 Department of Surgery, University of California, San Francisco

## Abstract

This cohort study examines measures of hospital interpreter usage for surgical patients with limited English proficiency (LEP) undergoing common general operations.

## Introduction

Disparities in surgical care among patients with limited English proficiency (LEP) are becoming increasingly well documented.^1,2,3^ This is especially concerning given that, as of 2021, 22% of the US population spoke a language other than English at home.^4^ Access to professional interpreters has been shown to increase surgical patients’ understanding of the indications and risks of their operation and discharge medication.^5^ Little is known, however, about factors within this vulnerable population that may affect which patients ultimately receive adequate access to perioperative interpretation. In this cohort study, we examine measures of hospital interpreter usage for surgical patients undergoing common general surgical operations.

## Methods

This study used electronic health record (EHR) data of patients aged 18 years or older who self-identified as having a preferred language other than English and were admitted for laparoscopic and/or open appendectomy, cholecystectomy, or colectomy at an urban academic center from 2019 to 2020. These data were a subset of data from our prior study^6^ using the same exclusion criteria, as well as those with missing data on covariates or outcomes. The institutional review board provided a waiver of participant consent because data were deidentified, in accordance with 45 CFR §46. This study followed STROBE reporting guidelines. The primary outcomes included documentation of interpreter usage within the first 24 hours of hospital encounter, interpreter usage at discharge, interpreter usage ever during this admission, and the provision of language-concordant discharge forms. Of note, interpreter documentation includes both in-person and telehealth, which are documented identically. Primary variables chosen a priori according to prior literature^6^ were self-identified race and ethnicity and language, gender, age, and insurance status. Data were analyzed from May to October 2021 using univariate analysis with Stata statistical software version 16.1 (StataCorp). Significance was set at 2-sided *P* < .05.

## Results

Of the 130 patients with LEP, the analytical cohort included 117 patients (74 female [63.3%]; mean [SD] age, 64 [17.3] years). Languages included Chinese (Cantonese, Mandarin, and Toishanese) languages (46 patients [39.3%]) and Spanish (34 patients [29.1%]), with the remaining 37 (31.6%) categorized as other, comprising 13 additional languages (Table 1).

**Table 1.** Characteristics of Patients

Characteristic	Patients, No. (%)
Age, mean (SD), y	63.8 (17.3)
Age range, y	
18-39	14 (12.0)
40-64	36 (30.8)
65-80	46 (39.3)
>80	21 (17.9)
Gender	
Female	74 (63.3)
Male	43 (36.8)
Race and ethnicity	
Asian	13 (11.1)
Latinx	36 (30.8)
White	63 (53.9)
Other^a^	5 (4.2)
Primary language	
Spanish	34 (29.1)
Chinese^b^	46 (39.3)
Other, non-English^c^	37 (31.6)
Insurance status	
Commercial	20 (17.1)
Medicare	62 (53.0)
Medicaid	33 (28.2)
Self	2 (1.7)
Length of stay, mean (SD), d	4 (1.6)

^a^ Includes Native Hawaiian or Other Pacific Islander and multiple races and ethnicities.

^b^ Includes Cantonese, Mandarin, and Toishanese.

^c^ Includes Russian, Vietnamese, Japanese, Tagalog, Arabic, Farsi, Korean, Punjabi, Bengali, Hindi, Tamil, Telugu, and Urdu.

Results on interpreter usage showed that 103 patients (88.3%) had interpreter use documented at least once throughout their length of stay (LOS), with 62 (53.0%) showing interpreter use within the first 24 hours and 4 (3.4%) at discharge. Overall, the study population had a mean (SD) of 1 (2) (median [IQR], 1 [1-2]) interpreter uses documented throughout their LOS. Only 14 patients with LEP (12.0%) were provided with language-concordant discharge forms (Table 2).

**Table 2.** Medical Record Review

Finding	Patients, No. (%)	*P* value
Documented interpreter uses during admission, No.		
Mean (SD)	1 (2)	NA
Median (IQR)	1 (1-2)	
Interpreter use		
At least once	103 (88.0)	NA
First 24 h	62 (53.0)	NA
At discharge	4 (3.4)	NA
Interpreter use first 24 h		
Other	1 [Reference]	NA
Chinese, OR (95% CI)	2.87 (1.17-7.05)^a^	.02
Spanish, OR (95% CI)	2.98 (1.13-7.84)^a^	.03
Language concordant discharge forms	14 (12.0)	NA

Abbreviations: NA, not applicable; OR, odds ratio.

^a^ Determined by univariate analysis.

Patients speaking Spanish or Chinese languages were approximately 3 times more likely than those speaking another language to have an interpreter used in the first 24 hours (Spanish, odds ratio, 2.87; 95% CI, 1.17-7.05, *P* = .02; Chinese language, odds ratio, 2.98; 95% CI, 1.13-7.84; *P* = .03), but these differences were not observed for any other of the assessed primary outcomes. Race and ethnicity, gender, age, and insurance status were not associated with interpreter use in univariate analyses (Table 2).

## Discussion

The findings of this cohort study indicate limited recorded interpreter usage for patients with LEP, suggesting the potential underutilization of available services. However, the inherent limitations in observational analyses of EHR-derived variables highlight the likelihood that actual interpreter usage exceeds documented instances. To establish a more reliable metric for assessing deficiency in language-concordant care, we propose examining the provision of language-concordant forms at discharge, which exist in the EHR only when provided to patients, and we found the rate to be exceptionally low. We also acknowledge the susceptibility of our study to self-reporting bias of language preference, because patients may underreport LEP on the basis of perceptions or social desirability. Further study of interpreter access would benefit from structured patient interviews regarding perioperative experience and pain management. In the interim, efforts to increase availability of language-concordant discharge forms provide critical opportunities to tangibly enhance the quality and understanding of both inpatient and postdischarge care plans of patients with LEP.

## Notes

Supplement.
Data Sharing Statement
