# Supplementary material for: Use of Professional Interpreters for Patients With Limited English Proficiency Undergoing Surgery
Source: JAMA Netw Open. 2024 Feb 6;7(2):e2355014. doi: 10.1001/jamanetworkopen.2023.55014 (PMC10848057; doi:10.1001/jamanetworkopen.2023.55014)
Supplement: Supplement. — Data Sharing Statement [file jamanetwopen-e2355014-s001.pdf]

Data Sharing Statement

Cevallos. Use of Professional Interpreters for Patients With Limited English Proficiency Undergoing Surgery. *JAMA Netw Open*. Published February 06, 2024. doi:10.1001/jamanetworkopen.2023.55014

Data
Data available: No
